# High and Sustained Participation in a Multi-year Voluntary Performance Measurement Initiative Among Primary Care Teams

**DOI:** 10.34172/ijhpm.2020.186

**Published:** 2020-10-19

**Authors:** Carol Mulder, Jennifer Rayner

**Affiliations:** ^1^Department of Family Medicine, Queens University, Kingston, ON, Canada.; ^2^Alliance for Healthier Communities, North York, ON, Canada.; ^3^Department of Medicine, Western University, London, ON, Canada.

**Keywords:** Primary Care, Performance Measurement, Ontario

## Abstract

**Background: **The province of Ontario, Canada has made major investments in interdisciplinary primary care teams. There is interest in both demonstrating and improving the quality of care they provide. Challenges include lack of consensus on the definition of quality and evidence that the process of measuring quality can be counter-productive to actually achieving it. This study describes how primary care teams in Ontario voluntarily measured quality at the team level.

**Methods:** Data for this 4-year observational study came from electronic medical records (EMRs), patient surveys and administrative reports. Descriptive statistics were calculated for individual measures (eg, access, preventive interventions) and composite indicators of quality and healthcare system costs. Repeated measures identified patient and practice characteristics related to quality and cost outcomes.

**Results:** Teams participated in an average of 5 of 8 possible iterations of the reporting process. There was variation between teams. For example, cervical cancer screening rates ranged from 21 to 86% of eligible patients. Rural teams had significantly better performance on some indicators (eg, continuity) and worse on others (eg, cancer screening). There were some statistical but small changes in performance over time.

**Conclusion:** High, sustained voluntary participation suggests that the initiative served a need for the primary care teams involved. The absence of robust data standards suggests that these standards were not crucial to achieve participation. The constant level of performance might mean that measurement has not yet led to improvement or that measures used might not accurately reflect improvement. The data reinforce the need to consider differences between rural and urban settings. They also suggest that further analysis is needed to identify characteristics that teams can change to improve the quality of care their patients experience. The study describes a practical, sustainable real-world approach to performance measurement in primary care that was attractive to interdisciplinary teams.

## Background

**Key Messages**

Implications for policy makers

- It is possible to measure quality in a sustained, voluntary way even without a universal definition of quality or mandated set of indicators. Given the importance of voluntary engagement in improving quality, a shift away from developing mandated reporting requirements towards increased consideration and support of voluntary initiatives may be in order.
- Participation in performance measurement might be less dependent on the robustness or completeness of the indicators used than the literature and current investment of effort might suggest.
- Rurality could be more explicitly considered when examining performance on quality indicators.

Implications for the public

It has been difficult to date to decide exactly how to measure the quality of care people get from primary care teams. Part of the challenge has been in agreeing what high-quality care is and then finding data to easily track progress with quality. This study tells the story of a group of primary care teams who went ahead anyway, even though they were not required to. They did it the best way they could, knowing it might not be perfect but would at least be a start. They found that it worked. Most of the group took part not just once but in as many as 8 cycles over 4 years. This shows that it is possible to move forward with tracking progress with quality even in the face of uncertainty about the perfect way to do it.

The province of Ontario, Canada has made major investments in the development of interdisciplinary primary care teams. Family Health Teams (FHTs), which are similar to the “patient medical home” concept in the United States and elsewhere^1^ were introduced in 2005. The intent was to capitalize on the promise of team-based care to generate better patient perceptions on important outcomes like access^2^ as well as clinical outcomes related to chronic disease management.^3^ In spite of this investment, Canada and Ontario have maintained a persistently poor showing on international comparisons of primary care quality.^4,5^There is also an increasing sense that primary care teams are too expensive.^6-8^ This study arose out of an interest in both demonstrating and improving the quality of care provided by interdisciplinary primary care teams.

 Part of the challenge in improving primary care is the difficulty in measuring quality. Firstly, there is a lack of consensus on the definition of quality. The Organisation for Economic Co-operation and Development and the Commonwealth Fund are two international organizations that routinely define and report national performance on quality of healthcare, including primary care. However, these frameworks and their associated indicators do not appear to work at the level of the individual provider or primary care team. For example, even though “access to an appointment on the same or next day” is part of the set of indicators commonly used in international comparisons of primary care quality,^5^ there is evidence that this measure is not meaningful to either patients or providers in Ontario.^9,10^ Deber and Schwartz^11^ describe similar issues with other measures that are commonly used but do not resonate with providers. For these or other reasons, many different visions and frameworks of indicators of primary care quality have emerged for intended use at the individual provider or team level.^12-15^This makes comparisons across settings regarding the impact of efforts to improve quality almost impossible.

 Another difficulty in primary care performance measurement is the increasing evidence that the process of measuring quality can be counter-productive to actually achieving it. Berwick,^16^ one of America’s most fervent evangelists for measurement and quality in healthcare from 1980 forwards, now says the solution to improving quality in healthcare is to *stop* excessively measuring. There is little evidence that measurement has improved quality.^14,17-19^ The reverse is starting to surface. Among the observed unintended negative results of performance measurement is the risk of increased inequity as providers preferentially focus on healthier patients who they feel are more likely to have good outcomes.^20^

 Cutting back on the number of indicators is not a solution. Focussing on a small number (or just one) indicator has been shown to detract attention from other aspects of primary care with resulting untoward clinical consequences.^21^ Limiting measurement to just a small number of measures also makes it harder to reflect the comprehensive, relationship-based nature of primary care.^13,14^ As Starfield (and many others) have observed, the true value of primary care does not lie in superior performance on specific clinical “body part” measures.^22,23^ Rather, it lies in the strength of the relationship between patients and their primary care providers,^20,24,25^ which in turn is based on the 4Cs: first Contact, Continuity, Comprehensiveness and Coordination of care.^26^ Talbot^27^ and Smith^28^ have noted that when people are judged on measures that they do not feel truly reflect the quality of their work, they can get demoralized, which tends to further undermine quality.

 Primary care teams want to demonstrate and continually improve the quality of care they provide. The Model for Improvement clearly positions measurement as a necessary element in efforts to improve.^29^ However, measuring quality is problematic. There are many indicators and frameworks of indicators but there is no standard definition of quality nor ideal way to measure quality at the practice or team level. Herein lies a dilemma: “Measurement is only a handmaiden to improvement but improvement cannot act without it.”^30^ This study therefore addresses the challenge of improvement by focussing on the process of measurement.

 This study describes the experience of Family Health Teams who were members of the Association of Family Health Teams of Ontario (AFHTO). AFHTO is a voluntary membership-based organization which represents nearly all of Ontario’s FHTs and some Nurse Practitioner-Led Clinics (NPLCs). All teams had administrative leadership and staff and used an electronic medical record (EMR). Some teams established formal partnerships to share Quality Improvement specialist resources. Depending on the team, the clinical staff complement included physicians, nurse-practitioners, nurses, social-workers, dietitians, pharmacists, occupational therapists or other interdisciplinary professionals. The mix of clinicians and the formal nature of their relationship with the administrative structure of the team varied between teams. Together, these teams provide care for approximately 3 million people (approximately 25% of the province’s population). Primary care is defined here as a community (vs hospital)-based ambulatory service. This is similar to the concept of “community-oriented primary care” described by the Institute of Medicine which is characterized as being integrated, comprehensive and based on sustained partnership with patients.^31^ One of AFHTO’s key strategic directions concerned demonstrating and further supporting improvements in the quality of team-based primary care. Hence, the interest of AFHTO in this study.

 The question this study addresses is “what does voluntary performance measurement look like in a collective of primary care teams in Ontario?” The study describes how primary care teams who were members of AFHTO voluntarily measured and reported their performance. It reports participation and performance across all teams and briefly explores differences between rural and urban teams to determine the need for stratification of performance analyses.

## Methods

 This longitudinal observational study took place over 4 years among primary care teams that were members of AFHTO.

###  Participants in the Measurement Initiative

 On behalf of its members, AFHTO implemented a performance measurement initiative called “Data to Decisions” (D2D) which produced a performance measurement report approximately every 6 months from 2014 to 2018. All members of AFHTO (approximately 192 organizations, depending on the year) were invited to participate in each of the 8 iterations of D2D. Participation was voluntary. Participation was evaluated through the number of teams contributing data and the number of indicators for which data were contributed.

###  Data Sources for the Measurement Initiative

 All data reported in D2D were submitted via a secure, web-based form to AFHTO by participating teams. All indicators were submitted at the team, vs individual patient, level. There was no patient-level data involved in D2D. The data came from three different sources: EMRs, patient surveys and administrative data reports. EMR data came from whichever EMR system was in place at the participating primary care team. This could be any of more than a dozen different systems in use in Ontario at the time of the study. Teams extracted data from their EMR according to guidance in a data dictionary publicly available via AFHTO’s website. Patient survey data came from surveys administered as part of normal operations within the team. Teams submitted data only for those survey questions that aligned with the wording in the data dictionary. Over time, some teams adjusted the wording of their patient surveys to increase their ability to submit data to D2D. The third source of data was an administrative data report^32^, definitions for which were published by the report’s producer, Health Quality Ontario. Because the administrative report was produced only for physicians, indicators based on data from these reports were not available to and therefore not submitted by NPLCs to D2D.

###  Indicators Included in the Measurement Initiative

 In keeping with the voluntary, member-driven nature of the initiative, the indicators included in D2D were selected by members through a modified Delphi process.^33,34^ These indicators covered common topics in primary care performance such as cancer screening, access and patient experience. D2D also included some novel indicators which are described in more detail here. The Diabetes composite score is an example of an indicator based on EMR data. The Diabetes composite score follows the lead of Minnesota and Wisconsin^35^ who were among the first to report a single metric reflecting several aspects of diabetes management. The version used here includes appropriate hemoglobin A1C (HbA1C) testing, appropriate HbA1C and blood pressure levels and cardiovascular protection via statin therapy, all based on clinical guidelines at the time. The score is presented as a percentage of the maximum possible total score.

*Per capita* healthcare system cost data is an example of an externally defined and calculated indicator sourced from administrative data reports.

 Quality was represented in D2D as a composite measure composed of 14 individual performance indicators. The composite quality score was based on the normalized performance of the individual components, each of which were weighted according to patients’ perception of the importance of the indicator in their relationship with their primary care provider.^36^ The composite quality score intentionally combined performance data on technical measures (eg, cancer screening) with measures of patient experience (eg, perception of courtesy of office staff) in an attempt to reflect a more comprehensive view of quality. The composite score is represented as a percentage of the maximum possible total score. More details of the composite are available on the AFHTO website.

 In addition to these performance indicators, teams provided data about their patient panel, self-reported status as a rural or urban team and teaching status.

###  Analysis of Participation and Performance Data

 Descriptive statistics were calculated for participation and performance on the indicators in the D2D reports. Data from the first iteration of D2D were excluded as some of the relevant indicators were not introduced until later. Differences in the number of indicators for which teams contributed data resulted in a different number of teams with complete data for each indicator. Random effect, random null model analyses were conducted using Mixed Linear Models procedures in SPSS^37^ to account for repeated measures in teams that contributed data for the same indicators in multiple iterations of D2D.

 Linear regression scores were calculated to describe performance in relationship to rurality, a characteristic important to the teams involved in the study. Linear regression scores were also used to describe performance over time. Sample sizes for linear regressions were a function of the number of teams with data for each performance indicator for each iteration and thus are higher than the total number of teams participating in D2D.

## Results

Table 1 describes the teams participating in at least one of the 8 iterations of D2D. It summarizes characteristics of the teams and their patient panels. Sample size for each characteristic varied because not all teams chose to provide data for all indicators in all iterations. The number of teams providing data for each element at least once in the eight iterations is indicated in the table.

**Table 1:** Characteristics of All Teams Participating in at Least One Iteration of D2D

**Characteristic**	**Estimate**	**95% CI**		**Number of Teams**	**Minimum**	**Maximum**
		**Lower Bound **	**Upper Bound **			
Mean number of D2D iterations per team	4.77	4.61	4.93	174	2	8
Rural (percent of teams)	41.82	35.25	48.38	174	n/a	n/a
Academic (percent of teams)	12.27	8.14	16.40	174	n/a	n/a
Mean patient panel size	19 772.86	16 233.15	23 312.57	150	813	197 994
Mean SAMI score	1.01	0.99	1.03	155	0.71	1.44
Hospital-EMR integration (percent of teams)	61.16	55.80	66.53	174	n/a	n/a

Abbreviations: EMR, electronic medical record; SAMI, Standardized Acute-Clinical-Groups Morbidity Index; D2D, Data to Decisions.

 Over the 4 years of the initiative, 174 teams contributed data to at least one iteration of D2D. On average, teams participated in 5 iterations. More than 60% of AFHTO members contributed to each of the iterations beyond the first two. Teams varied in patient panel size, setting, geographic location and teaching status.

Table 2 describes mean performance across all 8 iterations combined. It is limited to the indicators that were part of D2D in the eighth and most recent version. The descriptive statistics were calculated using repeated measures techniques to account for multiple data points of performance across multiple iterations. As with team characteristics, there was variation between teams. For example, the percent of eligible patients screened for cervical cancer in the time period covered by each iteration ranged from 21 to 86%. The smallest range for any of the percentage-type indicators was 47% (for patient involvement in decision-making).

**Table 2:** Mean Performance of All Participating Teams Over All Iterations of D2D, With Indicators in Order of Appearance in D2D

**Indicator (and Brief Definition -- Complete Definitions in Supplementary File 1)**	**Data Source**	**Mean**	**SE**	**95% CI**		**No. of Teams**	**Minimum**	**Maximum**
				**Lower Bound **	**Upper Bound **			
Patient-reported courtesy of office staff (% of survey respondents)	Patient survey	89.20	0.60	88.02	90.38	128	45.90	100.00
Patient-reported involvement in decision-making (% of survey respondents)	Patient survey	89.67	0.37	88.94	90.40	156	53.10	100.00
Colorectal cancer screening (% of eligible patients – indicator not available for NPLCs)	Admin. report*	67.26	0.60	66.07	68.45	161	26.10	84.00
Cervical cancer screening (% of eligible patients – indicator not available for NPLCs)	Admin. report	67.65	0.67	66.33	68.97	161	21.00	86.00
Childhood immunization (% immunized according to guidelines)	EMR	65.96	1.44	63.11	68.81	149	2.50	100.00
Diabetes management score (% of patients receiving care according to guidelines)	EMR	65.89	0.85	64.20	67.58	126	7.40	94.60
Patient reports a reasonable wait for appointment (% of survey respondents)	Patient survey	78.89	0.94	77.02	80.76	122	31.00	100.00
Patient reports ability to get appointment on same/next day (% of survey respondents)	Patient survey	56.53	2.66	51.27	61.78	155	7.14	100.00
Continuity (% of patients seeing own physician – not available for NPLCs)	Admin. report	66.27	0.90	64.50	68.04	157	13.50	90.40
Readmission within 30 days (% of patients with selected conditions – not available for NPLCs)	Admin. report	5.85	0.11	5.64	6.06	157	1.60	15.50
Total (adjusted) per capita cost (Canadian dollars per patient – not available for NPLCs)	Admin. report	2495.56	27.31	2441.45	2549.67	113	1643.07	4030.11
Overall quality score (% of maximum possible score)	Multiple	55.59	0.77	54.08	57.11	165	14.06	89.23

Abbreviations: EMR, electronic medical record; D2D, Data to Decisions; NPLCs, Nurse Practitioner-Led Clinics; SE, standard error. *Admin. Report: MyPractice, an administrative data report produced by Health Quality Ontario.

 Univariate correlation analyses of performance of rural and urban primary care teams are presented in Table 3. The direction of the differences is inferred from the sign of the T-value where negative values signify lower performance among rural teams. Table 3 shows that rurality was significantly related to performance on all but two of the performance indicators: readmissions and childhood immunization rates. The direction of the difference varied, with rural teams showing higher performance on some indicators (eg, continuity) but lower on others (eg, cancer screening). For this reason, subsequent correlations were stratified according to rurality.

**Table 3:** Differences in Performance Between Rural and Urban Primary Care Teams, With Statistically Significant Differences (*P* < .050) Indicated in Italics and Indicators Presented in Order of Appearance in D2D^a^

**Indicator (and Brief Definition – Complete Definitions in Supplementary File 1)**	**T Value**	**Significance (2-Tailed)**	**Mean Difference**	**95% CI**	
				**Lower Bound **	**Upper Bound **
*Patient-reported courtesy of office staff (% of survey respondents)*	2.74	.01	2.11	0.59	3.62
*Patient-reported involvement in decision-making (% of survey respondents)*	-2.19	.03	-1.09	-2.07	-0.11
*Colorectal cancer screening (% of eligible patients – indicator not available for NPLCs)*	-6.13	.00	-3.68	-4.85	-2.50
*Cervical cancer screening (% of eligible patients – indicator not available for NPLCs)*	-5.23	.00	-3.37	-4.64	-2.11
Childhood immunization (% immunized according to guidelines)	0.16	.88	0.27	-3.15	3.70
*Diabetes management score (% of patients receiving care according to guidelines)*	2.73	.01	2.90	0.81	4.98
*Patient reports a reasonable wait for appointment (% of survey respondents)*	-3.53	.00	-4.15	-6.45	-1.84
*Patient reports ability to get appointment on same/next day (% of survey respondents)*	-2.37	.02	-5.97	-10.92	-1.02
*Continuity (% of patients seeing own physician – not available for NPLCs)*	5.16	.00	5.05	3.13	6.98
Readmission within 30 days (% of patients with selected conditions *– *not available for NPLCs)	0.79	.43	0.14	-0.20	0.48
Total (adjusted) per capita cost (Canadian dollars per patient – not available for NPLCs)* (higher values less desirable)	2.19	.03	59.91	6.20	113.62
*Overall quality score (% of maximum possible score) *	-3.97	.00	-3.84	-5.74	-1.94

Abbreviations: D2D, Data to Decisions; NPLCs, Nurse Practitioner-Led Clinics. ^a^The direction of the differences is inferred from the sign of the T-value where negative values signify lower performance among rural teams.

Table 4 shows performance over time as measured through linear regression of the performance indicators against iterations of D2D. Coefficients for colorectal cancer screening and overall quality were statistically significant in both urban and rural teams, suggesting improvement over time. There were also statistically significant increases in performance for several other indicators but these differed between rural and urban teams. For example, there was a statistically significant increase in diabetes management performance in urban teams but not rural. In contrast, there was a statistically significant increase in continuity in rural teams but not urban. The practical significance of any of these observed improvements is questionable, however, since the coefficients are small and the R-squared values associated with the regression models are low, with none more than 0.06.

**Table 4:** Results of Univariate Linear Regressions of Individual D2D Indicators (Dependent Variable) on Iteration of D2D, Presented in Order of Appearance of Indicators in D2D

**Dependent Variable (and Brief Definition – Complete Definitions in Supplementary File 1)**	**Urban**				**Rural**			
	**N**	**Co-efficient**	***P*** ** Value**	**R-square**	**N**	**Co-efficient**	***P*** ** Value**	**R-square**
Patient-reported courtesy of office staff (% of survey respondents)	251	-0.135	.658	0.001	201	-0.614	.038	0.021
Patient-reported involvement in decision-making (% of survey respondents)	385	0.245	.184	0.005	302	0.196	.294	0.004
Colorectal cancer screening (% of eligible patients – indicator not available for NPLCs)	418	0.818	.000	0.059	338	1.038	.000	0.051
Cervical cancer screening (% of eligible patients – indicator not available for NPLCs)	401	-0.278	.146	0.005	334	0.051	.844	0.000
Childhood immunization (% immunized according to guidelines)	304	-0.718	.175	0.006	264	-1.520	.019	0.021
Diabetes management score (% of patients receiving care according to guidelines)	236	1.222	.010	0.028	218	0.283	.487	0.002
Patient reports a reasonable wait for appointment (% of survey respondents)	241	-1.083	.007	0.030	183	-0.389	.442	0.003
Patient reports ability to get appointment on same/next day (% of survey respondents)	374	-0.338	.397	0.002	284	1.023	.406	0.002
Continuity (% of patients seeing own physician – not available for NPLCs)	375	0.297	.312	0.003	313	0.992	.010	0.021
Readmission within 30 days (% of patients with selected conditions – not available for NPLCs)	405	0.141	.051	0.009	327	0.058	.164	0.006
Total (adjusted) per capita cost (Canadian dollars per patient – not available for NPLCs) (higher values less desirable)	289	4.992	.628	0.001	209	19.677	.101	0.013
Overall quality score (% of maximum possible score)	408	0.910	.009	0.017	374	0.623	.079	0.008

Abbreviations: D2D, Data to Decisions; NPLCs, Nurse Practitioner-Led Clinics.

## Discussion

 There was high and sustained voluntary participation in D2D, suggesting that some element of the process or content was serving a need for the primary care teams involved. At the very least, the pattern of participation illustrated that it is feasible to implement a performance measurement process on a voluntary, from-the-ground-up basis. Participation in measurement is important because measurement is a crucial element in improvement.^38^ Given that imposed measurement expectations tend to be poorly received,^13,27^ it is useful to see that measurement initiatives do not have to be mandated to have good uptake and thus support the improvement journey.

 The constant level of performance in the face of high and sustained participation in measurement could mean that measurement has not yet led to improvement in these teams. There are many possible reasons for this. Prime among them is the belief that change takes time and that even 4 years and 8 iterations might not have been sufficiently long to show improvement. In addition, not all teams participated over the entire time period, thus shortening the potential window for observing impact. Measurement error could also be a factor, as noted in the limitations section below. On the other hand, teams may have improved in areas not reflected in the indicators used here. In his warning that “It is wrong to suppose that if you can’t measure it, you can’t manage it,” Deming^39^suggested that the data that can be measured and reported may reflect some *but not all* of what matters in improving quality.^11^ For example, it may not be appropriate for a team focussing on youth mental health in response to local community issues to redirect their efforts to improving cervical cancer screening, even if their rate is lower than their peers. Participation in measurement is nonetheless valuable because it can expose assumptions,^40^ disrupt the status quo and thus prompt reflection and deliberate choices regarding improvement,^41^ even if they are not focussed on the variation illustrated in the data initially reported.

 The relationship between rurality and performance is consistent with other observations that expectations^42^ and delivery of primary care in rural areas are different from urban areas.^43^ These data do not address whether this is as it should be. However, they do suggest that stratification on the basis of rurality might be necessary to understand differences in performance in primary care across large and heterogeneous geographic areas. Currently, performance measurement reports in primary care, at least in Ontario, are not generally stratified according to rurality. These data add to the body of evidence calling for a change in that respect.

###  Limitations

 Quality as reported in this study is based on a limited number of indicators taken individually and collectively, in the form of a composite quality measure. The “individual indicator” approach suffers from the limitations attributed to ‘body part’ measures (described above). The composite indicator approach suffers from the limitations of most composite measures, most notably that it is hard for providers to know what to do to improve performance.^44^ Therefore, despite our best efforts, this vision of performance presented is still a very limited view of what primary care teams do.

 It is possible that the voluntary nature of data submission may have biased the sample towards inclusion of better performing teams. However, the eventual participation of a large proportion of the membership in the report suggests the risk of this is low. The risk that the data described here are not representative of the membership is further mitigated by the relatively high level of participation (ie, over 60% of teams in any one iteration with 70% in at least 3 iterations). Nonetheless, it is possible that the teams choosing to participate are different from their peers in important aspects affecting performance that are not reflected in these data.

 Measurement error is possible since all the data examined here were self-reported by teams. The risk of this was mitigated by intense engagement to ensure comparability of self-reported indicators between teams, something that was of primary interest to most participants. Nonetheless, teams may have diverged from the specifications in the data dictionary to better support their own quality improvement initiatives.

 The data are limited to the *FHT and NPLC* models currently deployed in Ontario, which cover about 25% of the primary care sector. Therefore, even if the data are representative of these teams, they cannot be considered to be representative of the entire sector.

## Conclusion

 We have described a practical, sustainable real-world approach to performance measurement in primary care in which teams voluntarily chose to participate. It succeeded in generating baseline data to support improvement efforts at the local (team) and collective (association) levels. The fact that this was observed in the absence of robust data quality standards suggests that while such standards may be important in understanding measurement data they were not, in this case, important in achieving *participation in* measurement.

 There is still a need to address outstanding questions about the impact of team characteristics and activities commonly understood to be enablers of quality. The observed relationship between rurality and performance suggest that the characteristics of teams can affect the quality of care their patients experience. Further analysis is needed to understand which characteristics are most tightly coupled to performance as well as how soon after implementation of performance measurement change in performance might be expected. Concrete data about the specific impact of characteristics that are within a team’s control to change can help primary care teams leverage their will and skill for performance measurement to improve quality.

## Acknowledgements

 We would like to thank AFHTO members for sharing your journey.

## Ethical issues

 This study approved by University of Toronto Research Ethics Board (protocol 31773).

## Competing interests

 CM received salary support from AFHTO for the duration of the initiative.

## Authors’ contributions

 CM: concept, design, implementation, data collection, analysis and writing. JR: design, implementation, analysis, writing.

## Authors’ affiliations

^1^Department of Family Medicine, Queens University, Kingston, ON, Canada. ^2^Alliance for Healthier Communities, North York, ON, Canada. ^3^Department of Medicine, Western University, London, ON, Canada.

## Supplementary files


Supplementary file 1. Sample Entry From Data Dictionary for Data to Decisions.
