# Supplementary material for: High and Sustained Participation in a Multi-year Voluntary Performance Measurement Initiative Among Primary Care Teams
Source: Int J Health Policy Manag. 2020 Oct 19;11(4):514–20. doi: 10.34172/ijhpm.2020.186 (PMC9309939; doi:10.34172/ijhpm.2020.186)
Supplement: Supplementary file 1 — Sample Entry From Data Dictionary for Data to Decisions. [file ijhpm-11-514-s001.pdf]

Supplementary file 1. Sample Entry From Data Dictionary for Data to Decisions

Cost		UPDATED
DESCRIPTION	**Indicator definition**	Per capita health care system cost with adjustment to reflect age/sex/complexity of patients*.<br>*Excludes palliative care patients.
	**Reference**	Primary Care Performance Measurement Framework, p. 221.<br>**For more information, see** Guidelines on Personal Level Costing (2013)
	**Type**	Outcome Indicator
	**External Alignment**	Primary Care Measurement Framework
DEFINTION & SOURCE INFORMATION	**Unit of analysis**	Per capita (per person)
	**Calculation**	For details of the calculation, see p. 53 of *MyPractice* Technical Appendix.<br>*Note*: We are linking directly to the HQO source as they are responsible for reporting any updates that may occur to how the indicator is calculated.<br>*Note:* The definition of this indicator has been changed for 2018 to exclude palliative care patients.
	**Data source**	*MyPractice* Team Report (ICES); see additional Excel worksheet (addendum to core report): *Cost*.<br>Access via HQO Portal
	**Data Elements**	• Total unadjusted Cost<br>• Adjusted Total Cost<br>• Unadjusted Primary Care Costs<br>• Unadjusted Physician, Lab, drug, ED and outpatient Costs<br>• Unadjusted Inpatient and same day surgery Costs<br>• Unadjusted Long-Term Care, Complex Continuing Care and Rehab Costs<br>**Note*: To be entered separately on D2D data submission form. Please see PCPMF reference for descriptions of each cost element.
OTHER RELEVANT INFORMATION	**Limitations/ Caveats**	Some teams might not have access to the *MyPractice* Team Report and therefore will not be able to report on this indicator. Those that have not signed up for the *MyPractice* Team Report may consider signing up via the HQO Portal.
	**Rationale**	Cost is one component of the Starfield Model triple aim for measuring primary care. The model states that one of the goals of comprehensive primary care is to support a sustainable health care system by reducing the total cost of care.
ADMIN	***Drafted on***	Nov. 17, 2015
	Drafted by	AFHTO Staff
	Updated on	June 23, 2016
	Updated by	AFHTO Staff
	Update history	Updated Data Elements to reflect current categories.
	Updated on	June 30, 2018
	Updated by	AFHTO Staff
	Update history	Palliative care exclusion
